# “Those Comments Last Forever”: Parents and Grandparents of Preschoolers Recount How They Became Aware of Their Own Body Weights as Children

**DOI:** 10.1371/journal.pone.0111974

**Published:** 2014-11-13

**Authors:** Karin Eli, Kyndal Howell, Philip A. Fisher, Paulina Nowicka

**Affiliations:** 1 Unit for Biocultural Variation and Obesity, Institute of Social and Cultural Anthropology, University of Oxford, Oxford, United Kingdom; 2 Department of Psychology, University of Oregon, Eugene, Oregon, United States of America; 3 Unit of Pediatrics, Department of Clinical Sciences, Intervention and Technology, Karolinska Institute, Stockholm, Sweden; University of Calgary, Canada

## Abstract

**Background:**

Parents' and grandparents' willingness to talk about children's body weights may be influenced by their own childhood experiences of body weight awareness and ‘weight talk’ in the family; however, little is known about how adults describe their recollected weight-related childhood experiences.

**Aims:**

This paper examines how parents and grandparents of preschoolers describe the emergence of their own body weight awareness in childhood or adolescence. The analysis highlights the sources that participants identify as having instigated their body weight awareness, the feelings and experiences participants associate with the experience of becoming aware of their body weights, and their framings of potential links between childhood experiences and attitudes and practices in adulthood.

**Methods:**

49 participants (22 parents, 27 grandparents, 70% women, 60% with overweight/obesity) from sixteen low-income families of children aged 3–5 years (50% girls, 56% with overweight/obesity) in the Pacific Northwest were interviewed. The interviews were videotaped, transcribed, and analyzed qualitatively.

**Results:**

Twenty-five participants (51%) said they became aware of their body weights in childhood or adolescence. Fourteen participants said their body weight awareness emerged through comments made by others, with the majority citing parents or peers. No participant described the emergence of body weight awareness in positive terms. Four participants directly linked their own negative experiences to the decision not to discuss body weight with their preschoolers. All four cited critical comments from their parents as instigating their own body weight awareness in childhood.

**Conclusions:**

In most cases, participants associated their emergent awareness of body weight with overtly negative feelings or consequences; some participants said these negative experiences continued to affect them as adults. Since family-based childhood obesity interventions involve open discussion of children's body sizes, the results suggest that clinicians should reframe the discussion to deconstruct obesity stigma and emphasize inclusive, affirmative, and health-focused messages.

## Introduction

Family-based interventions to prevent or manage obesity in children are most effective if implemented in early childhood [1]–[3]. An important part of these interventions is the engagement of family members in open discussion about children's body weights; however, parents are often reluctant to talk about their children's body weights with family members and with the children themselves. One potential reason for parental reluctance to discuss children's body weights is the pervasiveness of stigmatizing attitudes towards obesity [4]–[6], and the prevalence of negative ‘weight talk’ in the media [7]. As children are exposed to negative framings of overweight and obesity from an early age, both through the media and through peers [8], [9], parents and grandparents may view familial discussions of children's body weights as potentially reinforcing these negative framings.

Another reason for parents' and grandparents' reluctance to discuss children's body weights may be that they draw on their own childhood experiences of ‘weight talk’ in the family, or of becoming aware of their body weights [10]. Previous research has shown that parenting has an intergenerational dimension, such that parenting attitudes and practices are influenced by parents' own childhood experiences of home-life [11]. Other studies have also shown that parents have substantial influence on their children's body image and self-esteem in childhood and adolescence [10], [12], [13], as well as on their wellness practices, including healthy eating and engagement in physical activity [1], [14]–[16]. Moreover, as Bronfenbrenner argues, children's development is affected not only by the microlevel contexts of their immediate familial and biological environments, but also by mesolevel processes that bring the children's immediate environments into interaction, and exolevel contexts that do not involve the children directly [17]. Thus, parents' and grandparents' own body weight-related experiences with their families of origin or childhood peer groups may have important influence on their children and grandchildren. However, little is known about how adults, who are themselves parents and grandparents, recall their own experiences of becoming aware of their body weights as children or adolescents. As these experiences could influence discussions of children's body weights in family-based childhood obesity interventions, this paper aims to examine how parents and grandparents of preschoolers describe the emergence of their body weight awareness in childhood or adolescence. The analysis also highlights parents' and grandparents' framings of potential links between their childhood experiences of body weight awareness and their attitudes and practices as adults.

## Methods

### Ethics statement

The study was approved by the Internal Review Board of the Oregon Social Learning Center. At the visit, the informed consent forms were orally reviewed with parents and grandparents and questions were answered. If the parents/grandparents agreed to participate, they were asked to read and sign the written project description and project consent/permission forms. The families received a copy of the written study description and informed consent forms.

### Participants

Families of children aged 3–5 years from the Pacific Northwest (Eugene and Springfield metropolitan area, Oregon) were recruited in February – May 2011. The study's main research aim was to evaluate the role of grandparents in the development of preschoolers' lifestyles early in life, such that the active involvement of grandparents in family life (defined as spending time with the grandchild at least twice a month) was the primary criterion for inclusion in the study. Consequently, only families in which at least one parent and one grandparent were willing to be interviewed were included in the study. The other inclusion criteria specified that the child's age must be between 3–5 years, and that the child should have no underlying medical condition or disability which would affect his or her weight.

The participants were recruited through advertisements published in the job seekers' and volunteer sections of Craigslist and Register Guard, the local newspaper. The recruitment advertisements specified the study's inclusion criteria, with some advertisements targeting parents, and others targeting grandparents. Most advertisements contained a prompt related to children's body weights (e.g. “Do you think your child is overweight?, “Are you concerned about your child's weight?”, “Are you concerned that your grandchild is overweight?”), but did not include any reference to parents' or grandparents' body weights or body image concerns. The advertisements stated that each adult participant will receive $50 compensation for completing an interview. The recruitment advertisements are included as (Appendix S1).

In total, 49 family members (70% female) from sixteen families were interviewed. The participants' characteristics are summarized in Table 1. Due to the targeted recruitment process (ads in job advertisement sections) the sample displayed low levels of education and income; as many as 50% of parents were unemployed. Moreover, more than half of the parents and two thirds of the grandparents had overweight or obesity, according to WHO Body Mass Index (BMI) criteria (normal weight  = 18.5–24.9 kg/m^2^, overweight  = 25–29.9 kg/m^2^, obese ≥30 kg/m^2^) [18]. Of the children, 56% were either overweight or obese (overweight: 85^th^ percentile ≤BMI <95th percentile; obesity: BMI ≥95th percentile); those five who were categorized as obese were in the 95th, 96th, 98th (two children) and 99th percentiles for their BMI [19]–[21]. The majority of children, parents and grandparents were Caucasian, reflecting the ethnicity profile of this region of the Pacific Northwest.

**Table 1:** Descriptive statistics of the sample.

	Children (n = 16)	Parents (n = 22)	Grandparents (n = 27)
**Age** (mean in years, range):	4.6 (3.1–5.7)	32. 2 (22.7–49.5)	56.9 (43.0–77.9)
**Gender:**			
Female	8 (50%)	14 (64%)	21 (78%)
Male	8 (50%)	8 (36%)	6 (22%)
**Racial background:**			
Euro-American/Caucasian	11 (68%)	21 (95%)	23 (84%)
Native American	0	1 (5%)	0
Asian	0	0	1 (4%)
African-American	0	0	1 (4%)
Mixed	5 (32%)	0	2 (8%)
**BMI** (mean, range):	17.7 (14.3–21.5)	26.8 (16.1–39.1)	29.1 (16.1–49.4)
**Weight status:**			
Underweight	0	2 (9%)	1 (3%)
Normal weight	7 (44%)	8 (36%)	8 (30%)
Overweight	4 (25%)	6 (27%)	10 (37%)
Obese	5 (31%)	6 (27%)	8 (30%)
**Highest school grade completed:**	n/a		
High school		18 (82%)	20 (74%)
College/University		4 (18%)	7 (26%)
**Working situation:**	n/a		
Full time		7 (32%)	8 (30%)
Part time		4 (18%)	4 (15%)
Not employed*		11 (50%)	15 (55%)
**Annual household income**:	n/a		
Less than 14,999 USD		8 (36%)	7 (26%)
15,000–24,999 USD		6 (27%)	6 (22%)
25,000–39,999 USD		4 (18%)	6 (22%)
More than 40, 000 USD		4 (18%)	8 (30%)

*The main reasons for unemployment among parents were child care, pursuing higher education, and not finding work; among grandparents, unemployment was due to not finding work, reaching retirement age, or retiring due to health issues.

Parents and grandparents were interviewed separately at the Oregon Social Learning Center. Free child care was provided on site, and the children were not present during the interviews. Each interviewed participant received compensation of $50 for participating in the study. Prior to the interview, parents and grandparents completed a comprehensive sociodemographic questionnaire. All the interviewed parents and grandparents as well as the preschooler in focus had their height and weight measured, without shoes and wearing only light clothing, by trained research staff prior to the interviews. The measured weight status of the child or the parent/grandparent was not calculated before the interviews, and was not discussed with the parents or grandparents; thus, neither the interviewer nor the participants were told the weight statuses of the children and the interviewees. Although it was impossible to ensure blinding to participants' weight statuses (as some of the participants were aware of their own or their preschoolers' weight statuses, and as the researchers could observe that some interviewees were overweight), by not revealing measured weight statuses, the researchers were able to avoid placing the participants' BMI at the forefront of discussion.

The interviews were conducted by a single researcher, either the second author (who conducted approximately one third of the interviews) or the last author (who conducted approximately two thirds of the interviews). At the time of the study, the second author was a research assistant at the Oregon Social Learning Center (B.S. in psychology), with extensive background in working with preschoolers, and the last author was a postdoctoral researcher in psychology, with professional experience in family therapy. Each interview lasted 1.5–2.5 hours and explored the different roles of family members in shaping a child's lifestyle. The interviews followed a semi-structured schedule; while all participants were asked the same main questions, follow-up questions were tailored to individual responses. Most questions were open-ended and designed to facilitate detailed discussion. This paper focuses on the parents' and grandparents' accounts of the emergence of their own body weight awareness in childhood or adolescence. As such, the analysis examines interview extracts in which participants responded to the following main questions: “Did you think about your weight when you were a child, around 5–8 years of age?” and “At what age, if ever, did you start thinking about your weight?”

### Data analysis

The interviews were videotaped and transcribed in full. For this paper, transcript sections that related to the main questions were extracted, collated, and analyzed using a thematic analysis approach [22]. The analysis was specifically concerned with extracting themes that capture: (1) how participants describe the sources or processes that instigated their body weight awareness in childhood or adolescence, and (2) feelings or experiences that participants associate with the emergence of their body weight awareness in childhood or adolescence. A phenomenological orientation, which emphasizes participants' subjective experiences and the meanings they ascribe to them, guided the analysis [23]. A thematic analysis approach was selected, as the study aimed to identify patterns of recollected experience across the sample, rather than elucidate individual experiences on a case-by-case basis [22]. As participants' descriptive characterizations of experience and emotion were central to the study, the analysis also attended to the discourses participants used [23], while maintaining emphasis on experience.

The analysis included the transcript extracts of all participants who said they became aware of their body weights as children, and did not privilege the responses of participants whose preschoolers had overweight or obesity. This reflected the study's focus on explicating how parents and grandparents, in general, describe their own experiences of childhood body weight awareness, rather than on directly linking parents' and grandparents' recollected experiences to the weight statuses of their children or grandchildren. The analytic process started with an initial coding of the transcripts, which the first author, a postdoctoral researcher in medical anthropology, and the last author, a postdoctoral researcher in pediatrics, performed independently. The two coders then met to discuss the codes, examine and resolve potential disagreements, and decide on the clustering of codes into themes. To review the themes, the transcript extracts were revisited and organized by theme to create a detailed, quote-based, thematic map. The three thematic categories presented in this paper emerged from this thematic map. In constructing the thematic categories, the coders sought to elucidate the sources of body weight awareness, as described in the participants' accounts, and the emotional effects that participants associated with body weight awareness in childhood or adolescence.

## Results

Of the 49 participants, 25 (51%) said they became aware of their body weights in childhood or adolescence. Of these 25 participants, 68.0% had overweight or obesity, compared to 54.2% of the rest of the sample; this difference was not statistically significant (Fisher's Exact Test; p = 0.242). Fifteen of the 25 participants (60.0%) had a preschool aged child or grandchild with overweight or obesity, compared to 66.7% of the rest of the sample, a non-significant difference (Fisher's Exact Test; p = 0.426).

Fourteen participants said their body weight awareness emerged through comments made by others; four of the 14 added that their own childhood experience directly influenced their decision not to talk to their children or grandchildren about body weight. Eleven participants said their body weight awareness was self-initiated, with seven participants saying their awareness emerged through negative feelings about their body size or themselves, and four participants saying their awareness emerged through comparing themselves with peers. No participant described the emergence of body weight awareness in positive terms. Most participants (n = 15) associated the emergence of body weight awareness in childhood or adolescence with explicitly negative feelings or experiences. Compared to participants whose current weight status was underweight/normal weight, participants whose current weight status was overweight/obese were not more likely to recall childhood body weight experiences in negative terms (Fisher's Exact Test; p = 0.607). This suggests that adult weight status did not influence the negative or neutral framing of the participants' recollections. Salient excerpts from the transcripts of all 25 participants are included as Tables S1, S2, S3, S4, and S5.

### Other-initiated body weight awareness (Table S1)

Fourteen participants said their body weight awareness emerged through comments made by others, with the majority citing parents or peers. Two participants said they were teased about their weight by their classmates or siblings, and two others said they directly experienced critical comments from peers; however, body weight awareness also emerged through comments peers made about other children's bodies, or about themselves. As one participant described it: “I just remember my pants were getting tight, I remember other girls in my class were concerned about their weight. There would always be teasing, not to me, but to other heavier girls” (Gp14P1). Another participant related her emergent body weight awareness to the normative discussions that surrounded her at school, saying that “you're at an age where all the girls are like, ‘I'm fat! My thighs are so fat!’” (Gp01P1).

Seven of the participants linked the emergence of their body weight awareness to comments their parents made towards or about them. In two instances, participants described these comments with a neutral or mildly negative inflection: one participant said, “[m]y mom would point out when I was 9 that I would eat too much or that I needed to go exercise” (Gp12P2). In most cases, however, the comments the participants cited were overtly critical or judgmental. Parental criticism, as the participants described it, usually implicated the child's ‘excessively large’ size; for example, one participant said that, “I remember when I was about [my granddaughter's] age (…) I remember [mother] making me a costume and saying that my butt was too big for the pattern” (Gp01G3). Notably, participants who were considered ‘excessively skinny’ as children also said they were subjected to comments. In one case, a participant recounted that “I was really thin, and people constantly, constantly commented on my weight” (Gp06P1). In another case, a participant specifically cited her parents and other adult relatives as the source of commentary: “I was so skinny I was freaky. And my parents and relatives (…) [t]hey all said I was ugly” (Gp05G3).

### Self-initiated body weight awareness (Tables S2–S3)

Eleven participants described the emergence of body weight awareness as linked to their own feelings or thoughts about their bodies as children. Of those, seven participants said their awareness emerged through feelings about their body size or about themselves. One participant said she began to ‘feel fat’ after giving birth at 16 years old. The other participants, however, described such feelings as ongoing, with no definite starting points; for example, one participant said: “I don't think I've ever liked my body weight, even though I was only a size five up until I was 18 and that's fairly small, but I never liked myself” (Gp09G1). Likewise, another participant said: “I was very uncomfortable with my weight. From the time I can really remember. Yeah, from a very young age” (Gp07P1). When the researcher asked her, “did somebody tell you? I mean, how come you felt that you were different?”, the participant said, “I don't really remember anybody ever teasing me about it or telling me that I was fat. I just remember always feeling I was fat. And being preoccupied with it a lot” (Gp07P1). As this participant and others framed it, the sense of ‘feeling fat’ in childhood could emerge independently of (recollected) comments made by others.

In the accounts of the four other participants, body weight awareness emerged through comparisons with peers. For each participant, the comparison took a different turn. One woman said that, as a “tall skinny” adolescent, she longed to look like other, more shapely girls (Gp12G3), while another woman said that, throughout her adolescence, she felt other girls were “skinnier” (Gp01G1). The two men who said they compared themselves with peers noted concerns with body size and strength: one man said that, around age 10, he realized he was “a little chubby” and “bigger” than other children (Gp04P1), and another man said that he realized other boys were “stronger” following a fight with his friends (Gp02P1). While the participants' experiences were diverse, they all evoked a sense of difference compared to their peers.

### Associations of body weight awareness with negative feelings or experiences (Tables S4–S5)

No participant described the emergence of body weight awareness in positive terms. Ten participants described their experiences in neutral terms, with accounts centered on descriptive, rather than experiential, details, for example: “when I started developing and stuff she [mother] would nag me a little bit in high school about me gaining weight” (Gp04G1). However, most participants (n = 15) associated the emergence of body weight awareness in childhood or adolescence with explicitly negative feelings or experiences. In their accounts, some participants linked the emergence of body weight awareness with concomitant, persistent feelings of low self-esteem. For example, one participant said, “… I felt so fat. I really didn't like myself. It was really hard. I felt really awful about my weight” (Gp02G1), seamlessly linking feelings of ‘fatness’, weight dissatisfaction, and not liking oneself. Other participants linked the emergence of body weight awareness to teasing or critical comments, which they described as leading to feelings of inadequacy and inferiority as an immediate consequence. For example, one participant, now a grandmother, said her body weight awareness emerged when, at six years old, her mother expressed disappointment that she could not wear smaller children's clothes. She then reflected: “Well, I know now when I was six I wasn't heavy. (…) She [mother] thought they [smaller clothes] were cuter, but at six years old it made me think that there was something wrong with me, that I couldn't wear those clothes” (Gp03G1). This participant, like several others, added that these early experiences continued to affect her negatively as an adult.

A number of participants described the emergence of body weight awareness not only as influencing their self-esteem, but also as leading to negative behavioral consequences. Four of the participants related their emergent weight awareness to the development of eating disorders, either immediately or later in adolescence. One participant said she developed an eating disorder in the third grade: “I just remember always feeling I was fat. And being preoccupied with it a lot. (…) that's when I started making myself throw up and not eating” (Gp07P1). The other three participants said they developed eating disorders in adolescence, which they traced back to the emergence of their body weight awareness earlier in childhood. As one participant described it, “I had a very unhealthy image of what bodies should look like and I would exercise like four or five hours a day and I would limit my food or lie saying I ate” (Gp01P1).

Four participants directly linked their own negative experiences to the decision not to discuss body weight with their preschoolers; two of the participants had a grandchild with obesity, and the other two had a child or grandchild with normal weight. As these participants' preschoolers ranged in BMI status from normal weight to obesity, and given the small size of this sub-sample, these results do not imply a potential causal relationship between non-discussion of body weight and young children's BMI status. All four cited critical comments from their parents as instigating their own body weight awareness in childhood. One participant, now the grandmother of a preschooler with obesity, said: “I carry that with me today. I still think of that. (…) That's why it's so important to me not to make an issue out of weight, because those comments last forever and ever” (Gp01G3). Another participant, also the grandmother of a preschooler with obesity, said: “I would try and control it [the child's weight] at home, but not say anything, because it has impacted me my whole life” (Gp03G1). A participant who said she developed bulimia nervosa as an adolescent due to her father's repeated criticism of her body, explained that she decided to discuss body image positively with her children, rather than emphasize weight: “I'm really concerned having been a girl who had an eating disorder. I want her [my daughter] to grow up loving her body. Same thing for [my son]. Just honoring your body and taking care of it. We talk about that with the kids” (Gp04P2).

## Discussion

The findings demonstrate that when adults recall their own childhood experiences of becoming aware of their body weights, they tend to associate negative feelings or consequences with these experiences. While the participants spoke of different routes to body weight awareness – initiated by the comments of parents or peers, one's own ongoing negative body image, or comparisons with peers' bodies – no participant described becoming aware of her or his body weight as a positive experience. A number of participants said that their negative childhood experiences around body weight continued to affect their self-esteem as adults, and several participants added that these experiences influenced their decision not to discuss body weight with their own children or grandchildren. Notably, while most participants spoke of being overweight as children, some spoke of being ‘too skinny’; the central component of their experiences was the sense of having a dissatisfying body, one that diverged from the size considered normative or desirable.

Although some participants described their body weight awareness as having emerged independently of others' comments, for most, it was verbal feedback that instigated the process. This verbal feedback, which most participants attributed to parents or peers, could be direct, in the form of teasing or critical comments targeting the participants themselves, or indirect, in the form of peers commenting on their own body or on others' bodies. The salience of others' comments in this study is consistent with the well-documented association of critical comments, particularly those made by parents, with children's and adolescents' body dissatisfaction and low self-esteem [24]–[26].

Noticeably absent from the participants' accounts was discussion of possible media influences. Previous research has found that exposure to media images of ‘idealized’ bodies is associated with body dissatisfaction among adolescents [27] and that young women's exposure to media, without critical literacy, can lead to disordered eating behaviors [28]. As other studies have suggested, however, the media have limited influence on the body images of young children [9], and the influence of media on pre-adolescent children's body images may be indirect, with media messages gaining traction when disseminated through conversation with peers [8]. Thus, comments by parents and peers may act as influential mediators of broader sociocultural messages, and it is therefore possible that, for this study's participants, direct comments held a clearer causative role, as opposed to the indirect messages they (potentially) mediated.

Considered together, the participants' accounts demonstrated an overarching linking of body weight awareness, body dissatisfaction, and low self-esteem. With most participants citing others' comments as having led to their own body weight awareness, it is possible that parents and grandparents of preschoolers, through drawing on their own childhood experiences, may perceive the discussion of children's body weights as harmful to their body image and self-esteem. Notably, family-based interventions to manage obesity in early childhood involve open discussion of children's body sizes. This might impact the effectiveness of interventions, as parents and grandparents may also frame the discussions built into childhood obesity interventions as potentially harmful – with ‘weight talk’ perceived as more dangerous than obesity itself.

The results thus suggest that clinicians should ask parents and grandparents about their own experiences of body weight awareness in childhood, and explore sensitively and non-judgmentally how these experiences might have influenced their attitudes toward discussing childhood obesity. Moreover, childhood obesity interventions should reframe how families and clinicians discuss children's body sizes, placing the focus on wellbeing, not weight. Such reframing should employ inclusive and affirmative language to construct positive, health-focused messages, while directly opposing obesity stigma. As other researchers have suggested, messages that emphasize general wellness, physical activity, and healthy eating, rather than appearance, risk, and disease, can encourage children to adopt healthier lifestyles while protecting their self-esteem [29]–[33].

This study had some limitations. The study relied on adult participants' recollections of childhood experiences, and the results were thus subject to recall bias. The sample primarily consisted of families of Caucasian origin, representing the ethnic distribution of the population in Eugene and Springfield, Oregon. Thus, the influence of cultural background on childhood experiences of body weight awareness could not be investigated. Moreover, given the specific demographic characteristics of the sample, further research is needed to assess whether the results apply to other populations. In addition, as the questions focused on body weight awareness, rather than strength or size, they may not have captured the full extent and range of the male participants' experiences (see [34]); with only three men saying they were aware of their weight as children, further research is needed to examine gender-based differences in becoming aware of one's weight. Finally, although the interviews' open-ended approach allowed participants to make unbiased links between their past experiences and present-day parenting or grandparenting choices, directly questioning the participants about such links might have provided a richer understanding of the extent to which individuals' own childhood experiences influence their ‘weight talk’ with their children or grandchildren.

## Conclusion

This study examined how parents and grandparents of preschoolers describe the emergence of their own body weight awareness in childhood or adolescence. The analysis found that while participants cited various sources for their emergent awareness of body weight – including comments made by parents and peers, ongoing negative body image, and comparisons with peers – no participant associated this emergent awareness with positive experiences. Indeed, in most cases, participants associated their emergent awareness of body weight with overtly negative feelings or consequences; some participants said these negative experiences continued to affect them as adults, and several explicitly linked these negative experiences to their decision not to discuss body weight with their preschoolers. Since family-based interventions to manage obesity in early childhood require open discussion of children's body sizes, the results suggest that clinicians should ask parents and grandparents about their own experiences of body weight awareness in childhood, and explore how these experiences might influence their attitudes toward discussing childhood obesity. The results also suggest that childhood obesity interventions should reframe the discussion of children's body sizes among families and clinicians, directly deconstructing obesity stigma, and shifting towards inclusive, affirmative, and health-focused messages.

## Supporting Information

Table S1 **Awareness of body weight in childhood emerged through comments from parents or peers.**
(DOCX)

Table S2 **Awareness of body weight in childhood emerged through comparisons with peers.**
(DOCX)

Table S3 **Awareness of body weight in childhood emerged through negative feelings about one's own body size.**
(DOCX)

Table S4 **Awareness of body weight in childhood was associated with negative feelings or experiences.**
(DOCX)

Table S5 **Childhood experiences of comments on body weight directly influenced participants' approaches to discussing their preschoolers' body weights.**
(DOCX)

Appendix S1 **Recruitment advertisements in chronological order.**
(DOC)
