# Supplementary material for: “Those Comments Last Forever”: Parents and Grandparents of Preschoolers Recount How They Became Aware of Their Own Body Weights as Children
Source: PLoS One. 2014 Nov 13;9(11):e111974. doi: 10.1371/journal.pone.0111974 (PMC4230937; doi:10.1371/journal.pone.0111974)
Supplement: Table S1 — Awareness of body weight in childhood emerged through comments from parents or peers. (DOCX) [file pone.0111974.s001.docx]

**Table S1. Awareness of body weight in childhood emerged through comments from parents or peers.**

| 1. Gp01P1 (Mother) ***: you’re at that age where all the girls are like “I’m fat! My thighs are so fat! How can you stand there without your thighs touching?” (...) they would make comments on your weight or your looks or if you weren’t skinny enough to make it for cheerleading. |
| --- |
| 2. Gp04G1 (Grandmother, father’s mother) *: when I started developing and stuff she [mother] would nag me a little bit in high school about me gaining weight. And I don’t know quite why she did that because when I look at my pictures and I realize how big I was, I wasn’t that big. |
| 3. Gp06P1 (Mother) **: I was always really thin, and people constantly, constantly commented on my weight. I was really, really conscious of it. |
| 4. Gp07G1 (Grandmother, mother’s mother) *: my big sister told me I had a big butt. She called me “bubble butt.” And that stuck with me, when I was in junior high, even high school. |
| 5. Gp11P1 (Mother) ***: the one medication that actually worked to help my epilepsy made me eat a lot. (...) it didn’t really bother me until I did gain weight and I started getting made fun of at school. |
| 6. Gp12P1 (Mother) ***: I remember in high school, I broke my ankle and I remember somebody saying my butt was getting big. I think that was probably one of the first time I was like, okay. |
| 7. Gp12P2 (Father) ***: I started running when I was 9. My mom pointed out when I was 9 that I would eat too much or I needed to go exercise. |
| 8. Gp13P1 (Mother) ***: my dad always said stuff. (...) My mom told him I was getting boobs when I was 10, and he was like “Oh, that’s just fat.” Stuff like that, cause he’s really judgmental (...) So he’s been commenting about my weight constantly. My whole life. (...) I feel like he’s the one who gave me all my body image issues. |
| 9. Gp14P1 (Mother) **: When I got to either the 3rd or 4th grade, I started thinking about my weight. I just remember my pants were getting tight, I remember other girls in my class were concerned about their weight. There would always be teasing, not to me, but to other heavier girls. |
| 10. GP14G2 (Grandmother, father’s mother) **: I thought I was too heavy. My mom was really skinny and pretty, I was big boned and liked to eat. [Researcher: *Was it something that somebody said, or was it just the presence of people?*] Well, my brothers picked on me. Brothers can be cruel. |
| 11. Gp01G3 (Grandmother, father’s mother) ***: I remember when I was about [my granddaughter’s] age, I must have had a chunky behind because I remember my mom (…) telling me I was clumsy and that I needed to take dancing lessons because I was clumsy, but I remember her making me a costume and saying that my butt was too big for the pattern. And I remember being very self-conscious of that, even as a small child, and I carry that with me today. I still think of that. |
| 12. Gp04P2 (Mother) *: I remember, when I was 10 years old, was probably when I started to get overweight, and my parents helping me, if I lost 10 lbs I could get a new bicycle. I did, and that was totally unhealthy. I was diagnosed with bulimia [later], I had a lot of issues. |
| 13. Gp03G1 (Grandmother, mother’s mother) ***: My mother was really concerned about weight and appearance (…) She put me on a diet when I was 11 (…) I remember going to the movie theater, and instead she would send me with a hardboiled egg. And I remember canned asparagus; it was like a low carbohydrate diet I think. And her telling me when I was very small, I remember now what it is, being in JC Penny’s and she said, and I must have been six years old, she said, “Oh if only you could wear those cute clothes”. |
| 14. Gp05G3 (Grandmother, mother’s mother) *: I was so skinny I was kind of freaky. And my parents and relatives when we would be together, cause my sister was normal, she was a cute little, pudgy little girl (...) And my cousin as well, and I was super skinny. They all said I was ugly. [*Participant cries*] That [I thought] “What happened to me? How come they were so cute and I was so ugly?” |

Table legends: Gp# - family group number; P - parent; G – grandparent.

* = parent/grandparent of child with normal weight

** = parent/grandparent of child with overweight

*** = parent/grandparent of child with obesity
