# Supplementary material for: “Those Comments Last Forever”: Parents and Grandparents of Preschoolers Recount How They Became Aware of Their Own Body Weights as Children
Source: PLoS One. 2014 Nov 13;9(11):e111974. doi: 10.1371/journal.pone.0111974 (PMC4230937; doi:10.1371/journal.pone.0111974)
Supplement: Table S2 — Awareness of body weight in childhood emerged through comparisons with peers. (DOCX) [file pone.0111974.s002.docx]

**Table S2. Awareness of body weight in childhood emerged through comparisons to peers.**

| 1. Gp02P1 (Father) *: I remember, not like a straight angry fight, but me and my guy friends fighting and me being like, he’s stronger than me and realizing - but most of the people were a little bit older than me so I assumed that I’d get stronger as I get older. But not until I was 13-14 and started going to high school with weight training and stuff did I ever get conscious about what I weighed. |
| --- |
| 2. Gp01G1 (Grandmother, mother’s mother) ***: I started to think about it probably in high school. I thought things like that other girls were skinnier than me, but they probably weren’t. |
| 3. Gp04P1 (Father) *: It was late elementary school. I was in 4th or 5th grade. And in 4th grade I had mono, so I was probably 10 and I got a little chubby because I wasn't wrestling and I had to quit sports at the time. (…) I look back and I wasn’t ever heavy, I was just always bigger than the other kids. I was the tallest always. |
| 4. Gp12G3 (Grandmother, father’s mother) ***: It was more my shape than my weight. I was skinny. I just thought, “Why don’t I look like you?” I was this tall skinny girl. |

Table legends: Gp# - family group number; P - parent; G – grandparent.

* = parent/grandparent of child with normal weight

** = parent/grandparent of child with overweight

*** = parent/grandparent of child with obesity
