# Supplementary material for: “Those Comments Last Forever”: Parents and Grandparents of Preschoolers Recount How They Became Aware of Their Own Body Weights as Children
Source: PLoS One. 2014 Nov 13;9(11):e111974. doi: 10.1371/journal.pone.0111974 (PMC4230937; doi:10.1371/journal.pone.0111974)
Supplement: Table S3 — Awareness of body weight in childhood emerged through negative feelings about one's own body size. (DOCX) [file pone.0111974.s003.docx]

**Table S3. Awareness of body weight in childhood emerged through negative feelings about one’s own body size**

| 1. Gp02G1(Grandmother, father’s mother) *: I didn’t start thinking about my weight until after I had [my son] but I was only 16 when I had him and I remember feeling so fat (...) I really didn’t like myself. It was really hard. I felt really awful about my weight. |
| --- |
| 2. Gp07P1 (Mother) *: I was very uncomfortable with my weight. From the time I can really remember. Yeah, from a very young age. (…) I don’t really remember anybody ever teasing me about it or telling me that I was fat. I just remember always feeling I was fat. And being preoccupied with it a lot. |
| 3. Gp09G1 (Grandmother, mother’s mother) *: I don’t think I’ve ever liked my body weight, even though I was only a size five up until I was 18 and that’s fairly small, but I never liked myself. |
| 4. GP10G4 (Grandmother, stepmother of the father) **: I got my driver license when I was 16. And I [said I] weighed 100 pounds. But I only weighted 90 something. I was so skinny that [it] really bothered me. |
| 5. Gp12G2 (Grandfather, father’s father) ***: I’ve always been aware of my weight, because being small in stature and being younger, I was underweight, I was undersized. |
| 6. Gp13G1 (Grandmother, mother’s mother) ***: But I didn’t wear bathing suits, and shorts and things like that. I knew that I didn’t feel comfortable, but I was never. (…) But then I look back on my pictures and I really wasn’t all that fat, but in my brain I was fat. |
| 7. Gp15P1 (Mother) *: I was a cheerleader so I was in a little skirt and stuff, but I worked out so much. But I just remember feeling like, I always thought that I looked fat to myself but I never acted on it. |

Table legends: Gp# - family group number; P - parent; G – grandparent.

* = parent/grandparent of child with normal weight

** = parent/grandparent of child with overweight

*** = parent/grandparent of child with obesity
