# Supplementary material for: “Those Comments Last Forever”: Parents and Grandparents of Preschoolers Recount How They Became Aware of Their Own Body Weights as Children
Source: PLoS One. 2014 Nov 13;9(11):e111974. doi: 10.1371/journal.pone.0111974 (PMC4230937; doi:10.1371/journal.pone.0111974)
Supplement: Table S4 — Awareness of body weight in childhood was associated with negative feelings or experiences. (DOCX) [file pone.0111974.s004.docx]

**Table S4. Awareness of body weight in childhood was associated with negative feelings or experiences**

| 1. Gp01P1 (Mother) ***: I was crazy psycho about my weight. (…) I had a very unhealthy image of what bodies should look like and I would exercise like four or five hours a day and I would limit my food or lie saying I ate. |
| --- |
| 2. Gp07G1 (Grandmother, mother’s mother) *: my big sister told me I had a big butt (…)That was like when short jackets came in to style, and my winter coat came down to here [showing with hands, about mid-thigh] because I didn’t want anybody seeing my butt. (…) And so yeah I was really weight conscious. |
| 3. Gp11P1 (Mother) ***: I started getting made fun of at school. So then I was like, I didn’t even care because I didn’t have any friends at school and that’s when I started sitting in front of the TV all the time and my mom would just let me eat. |
| 4. Gp13P1 (Mother) ***: I remember my best friend and I being on diets in 6th grade. (…) But it got pretty bad, through the end of my high school years and into college, I started having really bad problems with bulimia and anorexia and stuff, so I was in treatment for that in college. Which they don’t even know about, my parents didn’t know about. |
| 5. GP14G2 (Grandmother, father’s mother) **: Well, my brothers picked on me, brothers can be cruel. [*Researcher:* *What age did you start thinking about your weight?*] I guess probably around 10 or 12, I was conscious of it before but it became consuming around that age because I put on some weight. |
| 6. Gp01G3 (Grandmother, father’s mother) ***: (…) I remember her [mother] making me a costume and saying that my butt was too big for the pattern. And I remember being very self-conscious of that, even as a small child, and I carry that with me today. |
| 7. Gp04P2 (Mother) *: I remember, when I was 10 years old, was probably when I started to get overweight, and my parents helping me, if I lost 10 lbs I could get a new bicycle. I did, and that was totally unhealthy. I was diagnosed with bulimia (later), I had a lot of issues. |
| 8. Gp03G1 (Grandmother, mother’s mother) ***: Well, I know now when I was six I wasn’t heavy. She [mother] was talking about when you jump from the size 6s and then you jump to the bigger girl’s clothes. She thought they were cuter, but at six years old it made me think that there was something wrong with me, that I couldn’t wear those clothes. |
| 9. Gp05G3 (Grandmother, mother’s mother) *: They all said I was ugly [*Participant cries*]. That [I thought] “What happened to me? How come they were so cute and I was so ugly?” |
| 10. Gp02G1(Grandmother, father’s mother) *: I was like 105 pounds when I got pregnant with him [my son] and after I had him and I lost weight I weighed about 115 pounds, which is not fat but I felt so fat. I really didn’t like myself. It was really hard. I felt really awful about my weight. |
| 11. Gp07P1 (Mother) *: I just remember always feeling I was fat. And being preoccupied with it a lot. (…) I mean I remember being in like the 3rd grade and I weighed, I was so obsessed with it, I weighed 62 pounds, and thought that I was just going, that’s when I started making myself throw up and not eating. |
| 12. Gp09G1 (Grandmother, mother’s mother) *: I don’t think I’ve ever liked my body weight. Even though I was only a size five up until I was 18 and that’s fairly small but I never liked myself. |
| 13. GP10G4 (Grandmother, stepmother of the father) **: I was so skinny that really bothered me. |
| 14. Gp12G3 (Grandmother, father’s mother) ***: It was more my shape than my weight. I was skinny. I just thought, “Why don’t I look like you?” I was this tall skinny girl. |
| 15. Gp13G1 (Grandmother, mother’s mother) ***: But I didn’t wear bathing suits, and shorts and things like that. I knew that I didn’t feel comfortable (…) |

Table legends: Gp# - family group number; P - parent; G – grandparent.

* = parent/grandparent of child with normal weight

** = parent/grandparent of child with overweight

*** = parent/grandparent of child with obesity
