# Supplementary material for: “Those Comments Last Forever”: Parents and Grandparents of Preschoolers Recount How They Became Aware of Their Own Body Weights as Children
Source: PLoS One. 2014 Nov 13;9(11):e111974. doi: 10.1371/journal.pone.0111974 (PMC4230937; doi:10.1371/journal.pone.0111974)
Supplement: Table S5 — Childhood experiences of comments on body weight directly influenced participants' approaches to discussing their preschoolers' body weights. (DOCX) [file pone.0111974.s005.docx]

**Table S5. Childhood experiences of comments on body weight directly influenced participants’ approaches to discussing their preschoolers’ body weights**

| 1. Gp01G3(Grandmother, father’s mother) ***: That’s why it’s so important to me not to make an issue out of weight, because those comments last forever and ever. |
| --- |
| 2. Gp04P2 (Mother) *: I’m really concerned having been a girl who had an eating disorder. I want her to grow up loving her body. Same thing for [my son]. Just honoring your body and taking care of it. We talk about that with the kids. (...) I think making kids feel good about their bodies is really important too. |
| 3. Gp03G1 (Grandmother, mother’s mother) ***: That is one of the reasons I swore I would never, never say anything about my children’s weight. I would try and control it at home, but not say anything, because it has impacted me my whole life. |
| 4. Gp05G3 (Grandmother, mother’s mother) *: They thought I was sleeping too, and I wasn’t. That’s why I tell [my daughter], “Shh,” while they’re asleep. You think they’re asleep, they’re not always asleep. |

Table legends: Gp# - family group number; P - parent; G – grandparent.

* = parent/grandparent of child with normal weight

** = parent/grandparent of child with overweight

*** = parent/grandparent of child with obesity
