# Supplementary material for: “Those Comments Last Forever”: Parents and Grandparents of Preschoolers Recount How They Became Aware of Their Own Body Weights as Children
Source: PLoS One. 2014 Nov 13;9(11):e111974. doi: 10.1371/journal.pone.0111974 (PMC4230937; doi:10.1371/journal.pone.0111974)
Supplement: Appendix S1 — Recruitment advertisements in chronological order. (DOC) [file pone.0111974.s006.doc]

**Appendix S1. Recruitment advertisements in chronological order**

**Craigslist, posted 26th January 2011 (et cetera jobs)**

*Are you concerned about your child’s weight? (Paid research study).*

Parents, do you have a child aged 3-5 years? Are you concerned about your child’s weight? If a grandparent is involved in your child’s life, you and the child's grandparent(s) may be eligible for a research study. Researchers at the Oregon Social Learning Center (OSLC) are interested in interviewing parent(s) and grandparent(s) about lifestyle choices in your family. Each family will receive $50 for a completed interview (it takes approximately 2 hours).

If you are interested in participating, please email Paulina at paulinan@oslc.org leaving your name and phone number to allow us to contact you by phone. However, please note that emails sent from some public email accounts such as gmail or yahoo may not be confidential.

Alternatively, if you prefer, you may call Kyndal 541-xxx-xxx ext. xxxx and leave your name and phone number.

**Group email to all employees at the Oregon Social Learning Center, 28^th^ of January 2011**

*Families with preschoolers needed to the Grandparents Study.*

Hi, we are looking for families with preschoolers with obesity to the Grandparents Study. We appreciate if you could forward this information to those who might be interested. Thank you! Paulina

*Are you concerned about your child’s weight? (Paid research study).*

Parents, do you have a child aged 3-5 years? Are you concerned about your child’s weight? If a grandparent is involved in your child’s life, you and the child's grandparent(s) may be eligible for a research study. Researchers at the Oregon Social Learning Center (OSLC) are interested in interviewing parent(s) and grandparent(s) about lifestyle choices in your family. Each family will receive $50 for a completed interview (it takes approximately 2 hours).

If you are interested in participating, please email Paulina at paulinan@oslc.org leaving your name and phone number to allow us to contact you by phone. However, please note that emails sent from some public email accounts such as gmail or yahoo may not be confidential.

Alternatively, if you prefer, you may call Paulina at 541-xxx-xx ext. xxxx or Kyndal 541-xxx-xxx ext. xxxx and leave your name and phone number.

**Craigslist, posted 19th February 2011 (et cetera jobs and volunteers)**

*Are you concerned that your grandchild is overweight? Paid study (Eugene)*

Grandparents, do you have a grandchild aged 3-5 years? Are you concerned that your grandchild is overweight? If you are involved in your grandchild’s life, you and the grandchild's parent(s) may be eligible for a research study. Researchers at the Oregon Social Learning Center (OSLC) are interested in interviewing parent(s) and grandparent(s) about lifestyle choices in families with young children. Each family member will receive $50 for a completed interview (approximately 2 hours).

If you are interested in participating, please call Kyndal 541-xxx-xxxx ext. xxxx and leave your name and phone number.

**Ad in the Register Guard, daily newspaper, 27^th^ February 2011 and 1^st^ of March 2011**

GRANDPARENTS: Are you concerned that your grandchild is overweight? Do you have a grandchild aged 3-5 years old? If you are involved in your grandchild’s life, you & the grandchild’s parent(s) may be eligible for a research study. Each family member will receive receive $50/interview. Please call Kyndal, Oregon Social Learning Ctr 541-xxx-xxxx ext. xxxx.

**Craigslist, posted 5th March 2011 (volunteers and et cetera jobs)**

*Are you concerned that your child is overweight? (Paid research study) (Eugene)*

Parents, do you have a child aged 3-5 years? Are you concerned that your child is overweight? If a grandparent is involved in your child’s life, you and the child's grandparent(s) may be eligible for a research study. Researchers at the Oregon Social Learning Center (OSLC) are interested in interviewing parent(s) and grandparent(s) about lifestyle choices in your family. Each family member will receive $50 for a completed interview (it takes approximately 2 hours).

If you are interested in participating, please call Kyndal 541-485-2711 ext. 1390 and leave your name and phone number. If you are interested in participating, please call Kyndal 541-xxx-xxxx ext. xxxx and leave your name and phone number.

**Craigslist, posted 27th March 2011 (et cetera jobs)**

*Do you think that your child is overweight? (Paid research study) (Eugene)*

Parents, do you have a child aged 3-5 years? Do you think that your child is overweight? If a grandparent is involved in your child’s life, you and the child's grandparent(s) may be eligible for a research study. Researchers at the Oregon Social Learning Center (OSLC) are interested in interviewing parent(s) and grandparent(s) about lifestyle choices in your family. Each family member will receive $50 for a completed interview (1.5 h). We provide child care during the interview.

If you are interested in participating, please call Kyndal 541-xxx-xxxx ext. xxxx and leave your name and phone number.

**Craigslist, posted 30th March 2011 (et cetera jobs)**

*Is your grandchild a little pudgy? (Paid research study)* *(Eugene)*

Grandparents, do you have a grandchild aged 3-5 years? Is your grandchild a little pudgy? If you are involved in your grandchild’s life, you and the child's parent(s) may be eligible for a research study. Researchers at the Oregon Social Learning Center (OSLC) are interested in interviewing parent(s) and grandparent(s) about lifestyle choices in your family. Each family member (parents and grandparents) will receive $50 for a completed interview (1.5 h). We provide child care during the interview.

If you are interested in participating, please call Kyndal 541-xxx-xxxx ext. xxxx and leave your name and phone number.

Compensation: Each family member will receive $50 for a completed interview (1.5 h). We provide child care during the interview.

Non-profit organization

**Craigslist, posted 30th March 2011 (volunteers)**

*Is your grandchild is a little pudgy? (Paid research study) (Eugene)*

Grandparents, do you have a grandchild aged 3-5 years? Is your grandchild a little pudgy? If you are involved in your grandchild’s life, you and the child's parent(s) may be eligible for a research study. Researchers at the Oregon Social Learning Center (OSLC) are interested in interviewing parent(s) and grandparent(s) about lifestyle choices in your family. Each family member will receive $50 for a completed interview (1.5 h). We provide child care during the interview.

If you are interested in participating, please call Kyndal 541-xxx-xxxx ext. xxxx and leave your name and phone number.

**Craigslist, posted 28th April 2011**

*Is your child bigger than other children? (Paid research study)* *(Eugene)*

Parents, do you have a child aged 3-5 years? Are you concerned that your child is overweight? If a grandparent is involved in your child’s life, you and the child's grandparent(s) may be eligible for a research study. Researchers at the Oregon Social Learning Center (OSLC) are interested in interviewing parent(s) and grandparent(s) about lifestyle choices in your family. Each family member will receive $50 for a completed interview (1.5 h). We provide child care during the interview.

If you are interested in participating, please call Kyndal 541-xxx-xxxx ext. xxxx and leave your name and phone number.

Compensation: Each family member will receive $50 for a completed interview (1.5 h). We provide child care during the interview.

Non-profit organization

**Craigslist, posted 13th May 2011 (et cetera jobs)**

*Looking for big preschoolers! (Paid research study)* *(Eugene)*

Parents, do you have a child aged 3-5 years? Are you concerned that your child is overweight? If a grandparent is involved in your child’s life, you and the child's grandparent(s) may be eligible for a research study. Researchers at the Oregon Social Learning Center (OSLC) are interested in interviewing parent(s) and grandparent(s) about lifestyle choices in your family. Each family member will receive $50 for a completed interview (1.5 h). We provide child care during the interview.

If you are interested in participating, please call Kyndal 541-xxx-xxxx ext. xxxx and leave your name and phone number.

Compensation: Each family member will receive $50 for a completed interview (1.5 h). We provide child care during the interview.

Non-profit organization
